# Preservation of amorphous ultrafine material: A proposed proxy for slip during recent earthquakes on active faults

**DOI:** 10.1038/srep36536

**Published:** 2016-11-09

**Authors:** Tetsuro Hirono, Satoru Asayama, Shunya Kaneki, Akihiro Ito

**Affiliations:** 1 Department of Earth and Space Science, Graduate School of Science, Osaka University, Toyonaka, Osaka 560-0043, Japan; 2 Analytical Instrument Facility, Graduate School of Science, Osaka University, Toyonaka, Osaka 560-0043, Japan

## Abstract

The criteria for designating an “Active Fault” not only are important for understanding regional tectonics, but also are a paramount issue for assessing the earthquake risk of faults that are near important structures such as nuclear power plants. Here we propose a proxy, based on the preservation of amorphous ultrafine particles, to assess fault activity within the last millennium. X-ray diffraction data and electron microscope observations of samples from an active fault demonstrated the preservation of large amounts of amorphous ultrafine particles in two slip zones that last ruptured in 1596 and 1999, respectively. A chemical kinetic evaluation of the dissolution process indicated that such particles could survive for centuries, which is consistent with the observations. Thus, preservation of amorphous ultrafine particles in a fault may be valuable for assessing the fault’s latest activity, aiding efforts to evaluate faults that may damage critical facilities in tectonically active zones.

Japan is subject to frequent earthquakes, and active faults are abundant not only along offshore plate subduction boundaries but also inland (Fig. 1a). The earthquake risk to nuclear power plants in Japan was a matter of debate before the 2011 Tohoku-oki earthquake, notably after the 2007 M_w_ 6.6 Niigata Chuetsu-oki earthquake caused minor damage to the Kashiwazaki-Kariwa power plant and the release of a negligible amount of radiation^1^. The earthquake risk of the Chubu active fault system was also considered in 2000^2^. However, since the Fukushima Daiichi nuclear power plant disaster in 2011, most nuclear power plants in Japan have been shut down and their seismic safety is being reassessed. A prime concern is whether mapped faults near a plant are active; for example, the Tsuruga plant’s reactors are 250 m from a known active fault, and several minor faults may run beneath the reactors^3^,^4^.

The debate centres on the recent activity of faults near the plants, as recognized in the criteria for formally designating an “Active Fault”. The currently preferred evaluation standard is the displacement by faulting of Quaternary strata of known sedimentary age^5^, but it is not feasible where suitable material was removed during plant construction and no nearby outcrops of the fault are available. Another potential proxy for recent fault activity was proposed after the discovery of ultrafine particles (partly amorphous) on the Chelungpu fault in the slip zone of the 1999 Chi-Chi earthquake in Taiwan^6^. As such particles, produced by comminution during the earthquake^6^, are easily dissolved into the interstitial fluid^7^,^8^, their preservation in a slip zone would mean that ruptures on the fault are recent. Because more investigations in various types of fault rock are needed to confirm the proxy^6^, it was noteworthy when another example of ultrafine particles in an active fault was discovered in fault gouge on the Median Tectonic Line (MTL), Japan^9^. The Chelungpu fault and the MTL intersect sedimentary and metamorphic rocks, respectively. However, another crucial analytical task is to assess the durability of these chemically unstable ultrafine particles. Thus we chose to focus on the lifetime of ultrafine particles under the near-surface environmental conditions found within faults.

The ultrafine particles in the slip zone may be expected to dissolve in interstitial fluids during the interseismic period. To explore this process, we targeted the ultrafine particles in the Chelungpu fault and in the Arima-Takatsuki Tectonic Line (ATTL, Fig. 1b), which last slipped during the 1596 Keicho-Fushimi earthquake (M ~ 7.5)^10^. We first quantified the amount of amorphous ultrafine particles in these active slip zones, and then established the mineralogical and crystallographic nature of these particles. We next evaluated the duration of these particles, in both the ATTL and the Chelungpu fault, by using the chemical kinetics of the dissolution reaction to establish an indicator for recent fault activity.

## Methodological procedure

We tested a new approach consisting of the following four steps: (1) Determine the amount of the amorphous component both in and around the fault and conduct microscopic observations of the ultrafine particles. (2) Determine whether the ultrafine particles are crystalline or amorphous and analyse their atomic composition. (3) Characterize the environment in which the ultrafine particles formed (near-surface or hydrothermal) on the basis of their nanometric structure and *in-situ* environmental conditions such as temperature and pH. (4) Perform chemical kinetic modelling of the dissolution process to estimate the lifetime of amorphous ultrafine particles, thus constraining the most recent time that the fault slipped.

### Geological background

**Figure 1:**
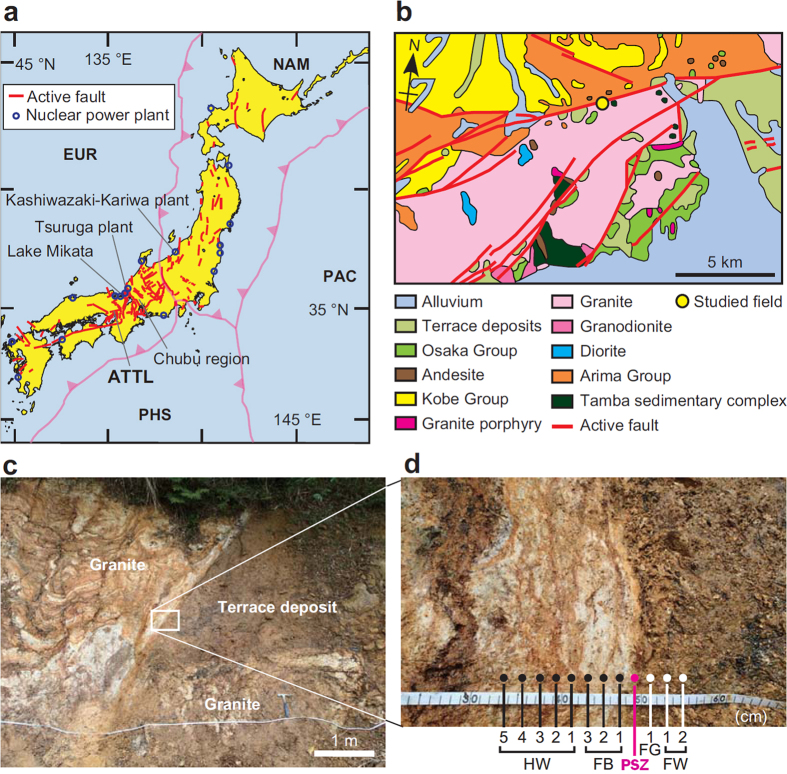
Locations of nuclear power plants and active faults in Japan. (**a**) Tectonic setting of the Japanese islands with locations of nuclear plants, major active faults, and features named in the text. EUR, Eurasia plate; NAM, North America plate; PHS, Philippine Sea plate; PAC, Pacific plate. This map was modified by the authors from open-source data^53^ using Adobe Illustrator graphic software. (**b**) Geologic map around the ATTL, modified by the authors from published data^10^ using Adobe Illustrator. (**c**) Photo of study site showing displacement between a Quaternary terrace deposit and Cretaceous granite along the ATTL. (**d**) Close-up of the ATTL fault zone showing sampling points. HW, hanging wall; FB, fault breccia; PSZ, primary slip zone; FG, fault gouge; FW, footwall.

The ATTL has a predominantly right-lateral strike-slip sense of displacement^10^. A fault outcrop near Arima exposes a fluvial terrace with a ^14^C age of 7870 ± 14 years before present (BP) and Cretaceous granite (Fig. 1c), both of which display a vertical offset of 2.1 m along the ATTL^10^. Displacement at this locality during the 1596 earthquake is supported by the similar displacement of fluvial deposits with a ^14^C age of 580 ± 40 years BP at a nearby outcrop^10^. The fault outcrop displays a fault zone consisting of a fault breccia 7 cm thick and a layer of very fragile fault gouge 5 cm thick that includes a sharply defined surface, identified as the primary slip zone (PSZ) (Fig. 1d). Fluid seeping from the fault zone probably originated from the local meteoric water. We collected 12 samples from the PSZ and the surrounding material for the following analyses and observations.

A borehole core sample of the Chelungpu fault was recovered from the Chinshui shale, of early Pliocene age, by the Taiwan Chelungpu-fault Drilling Project (Supplementary Figure 1). Three major zones of faulting within the Chinshui shale have been interpreted as segments of the Chelungpu fault^11^,^12^, and the shallowest of these is most likely the one that slipped during the Chi-Chi earthquake, because recent heating and a major anomaly in stress orientation were observed in that zone^13^,^14^,^15^. The PSZ of the Chi-Chi earthquake was identified as a millimetre-thick shear zone within black gouge, because newly formed magnetite in the gouge carried a stable palaeomagnetic component consistent with the 1999 reference geomagnetic field^16^. Fluid flow along the fault zone was detected by borehole hydraulic measurements^17^,^18^, and isotopic evidence suggested that the fluid originates from surface meteoric water and a deep crustal source^19^. For this study we examined six samples from the PSZ and the surrounding materials that were used in a previous analysis^6^.

### Quantification of amorphous ultrafine particles

**Figure 2:**
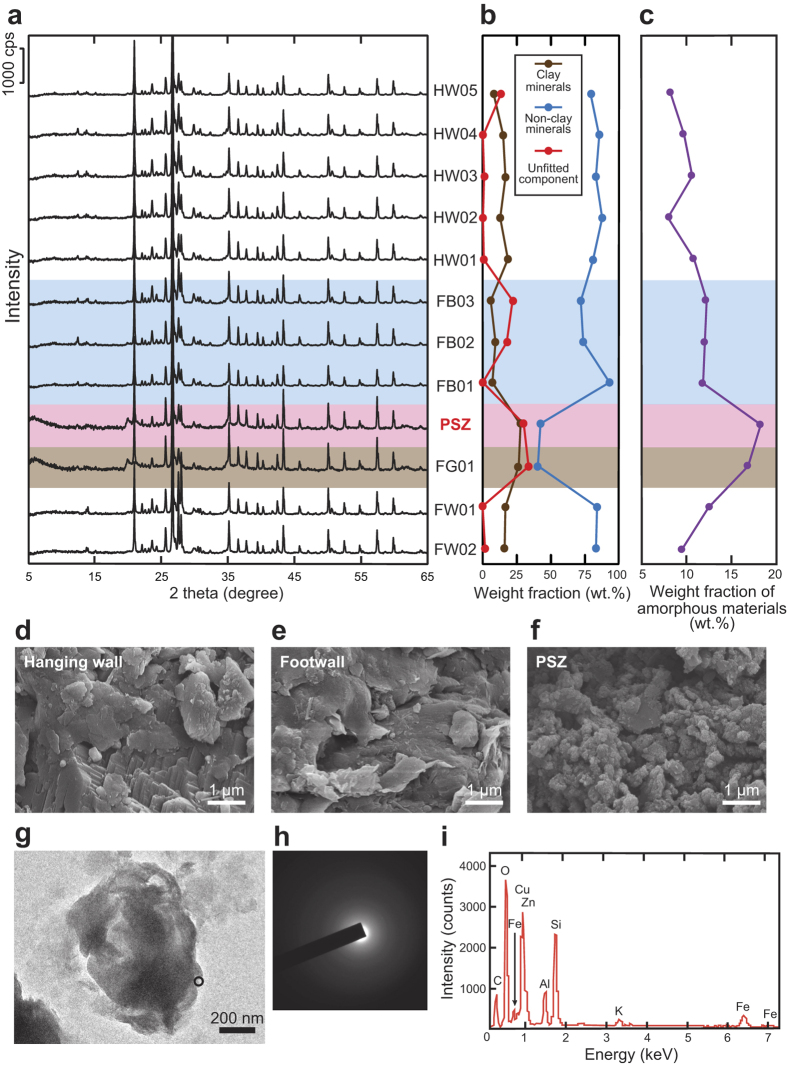
Data and images of amorphous ultrafine particles preserved in the PSZ of the ATTL. (**a**) X-ray diffraction patterns of all samples (see Method). (**b**) Weight fractions of clay minerals, non-clay minerals and component not fitted by RockJock program. (**c**) Weight fraction of amorphous materials determined by standard curve for the integral XRD intensity versus the amount. (**d–f**) Scanning electron micrographs of material from the hanging wall, footwall and PSZ, respectively. (**g**) Bright-field transmission electron micrograph of ultrafine particles from the PSZ. (**h**) Electron diffraction pattern of the particle within the circle in **g**. (**i**) Energy dispersive X-ray signals from the particle from the PSZ.

**Figure 3:**
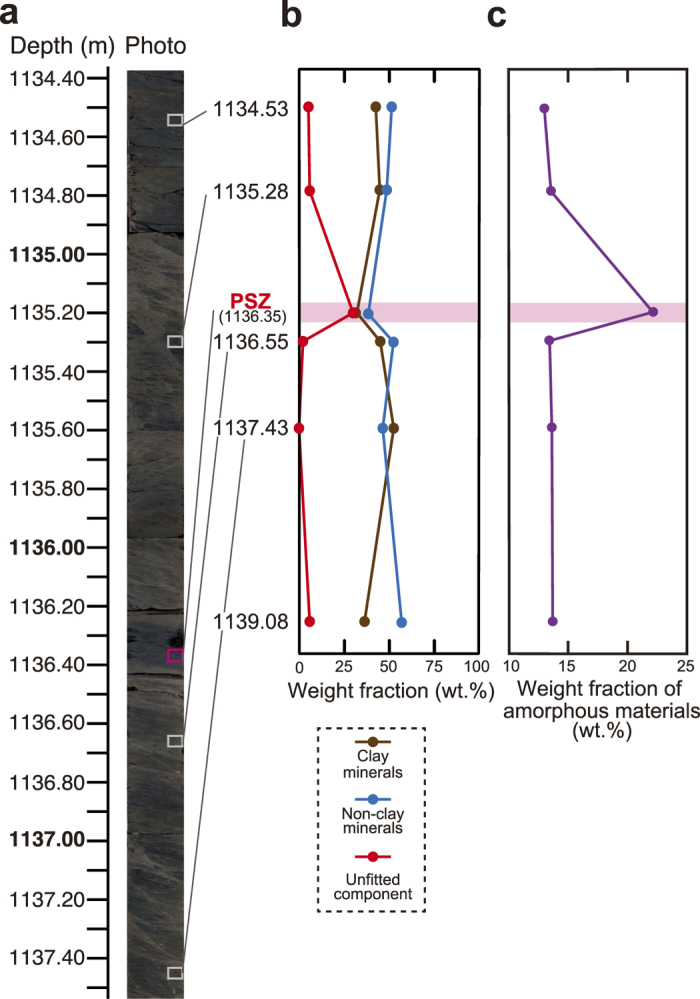
Data of amorphous ultrafine particles preserved in the PSZ of the Chelungpu fault. (**a**) Photo of the borehole core sample. Small boxes in the photo indicate the sampling points, except for the sample at 1139.08 m depth. (**b**) Weight fractions of clay minerals, non-clay minerals and component not fitted by RockJock program (data from ref. 6). (**c**) Weight fraction of amorphous materials determined by standard curve for the integral XRD intensity versus the amount.

Mineral phases and their modal amounts were determined by X-ray diffraction (XRD) using the RockJock program^20^ to fit the diffraction data to the patterns of standard minerals (Fig. 2a,b; see Methods). The fault gouge and PSZ samples from the ATTL were identified as predominantly clay minerals and unfitted components, along with relatively small amounts of non-clay minerals such as quartz. The unfitted components were attributed to amorphous ultrafine materials rather than to minerals that were not identified in RockJock. In previous analyses of the PSZ and surrounding host materials of the Chelungpu fault (Fig. 3a,b; also see Fig. 2 in ref. 6), the unfitted components were also attributed to amorphous ultrafine materials^6^. The standard deviation of total mineral amounts (and unfitted components) from these RockJock results was reported as ±5.9 wt.%^6^.

To test this attribution and attempt to quantify the amount of amorphous material in the samples, we obtained XRD spectra of prepared mixtures of amorphous silica (Kanto Chemical, Japan) and quartz powder (Wako Pure Chemical Industries, Japan) by using a zero-diffraction plate (made from a single silicon crystal). Sharp peaks in the XRD spectra of these mixtures were attributed to crystalline quartz and the internal standard (α-alumina), but wider, more subdued peaks were observed around 20–30° 2θ, indicating amorphous material^21^,^22^, in samples with smaller quartz contents (Supplementary Figure 2a). Most silicate minerals, such as feldspar, show similar broad bump in their amorphous state^23^. After subtracting the crystalline peaks and the background curve from the XRD patterns by using multivariate analysis (with expressions for position, height and width of crystalline peaks and the background curve), we found the integral intensities of the amorphous peaks to be inversely proportional to the weight fraction of crystalline components (Fig. 2b) and were thereby able to quantify the complementary fraction of amorphous components in the samples. Triplicate multivariate analyses of the XRD spectra from two separate samples (six analyses in total) showed that determinations of the amount of the amorphous component had a standard deviation of ±1.2 wt.%.

## Results

### Amounts of amorphous ultrafine particles in active faults

In the case of the ATTL, the PSZ samples contained the largest proportion of inferred amorphous material (18.2 wt.%), and the surrounding rock samples averaged 11.1 wt.% amorphous material (Fig. 2c). Likewise for the Chelungpu fault, the PSZ samples contained the largest proportion of inferred amorphous material (22.1 wt.%), and the surrounding rock samples averaged 13.6 wt.% amorphous material (Fig. 3c).

**Figure 4:**
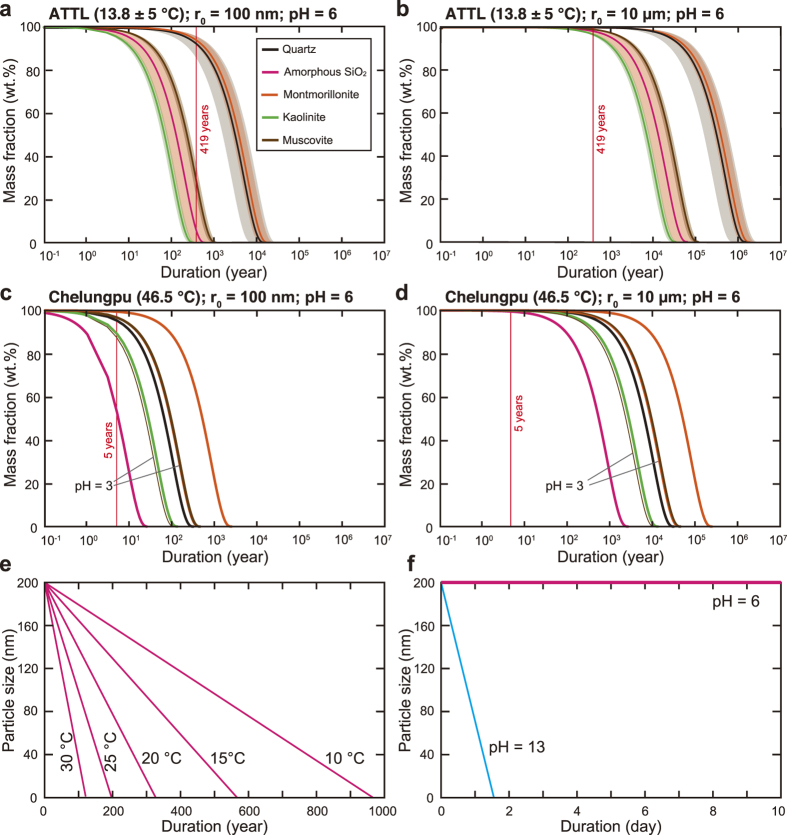
Changes in mass fraction and size of spherical particles with time. (**a**,**b**) Change in mass fractions of single particles of 100 nm and 10 μm initial radii, respectively, for the ATTL. (**c**,**d**) Change in mass fractions of single particles of 100 nm and 10 μm initial radii, respectively, for the Chelungpu fault. (**e**) Change with time in the particle size of amorphous silica at different temperatures. Initial radius of the particle is 100 nm. (**f**) Change with time in the particle size of amorphous silica at pH 6 and 13.

Under the electron microscope, the host rocks of the ATTL exhibited structures typical of sedimentary rock and consisted mainly of intact quartz and feldspar particles several to several tens of micrometres in diameter (Fig. 2d,e), whereas the PSZ of the ATTL contained single and aggregated, well-rounded ultrafine particles several tens to several hundreds of nanometres in size (Fig. 2f,g; see Methods). The diffuse scattering exhibited by selected portions of individual particles in one of these aggregates (Fig. 2g) suggests a noncrystalline structure (Fig. 2h), and the energy dispersive X-ray spectrum of the aggregate is consistent with that of clay minerals (Fig. 2i). Similar amorphous ultrafine particles were observed in the PSZ of the Chelungpu fault (Figs 4 and 5 in ref. 6).

The transformation of crystalline mineral phases into amorphous material and the formation of ultrafine particles may result from lattice distortion and granulation by coseismic slip, as has been demonstrated by friction and milling experiments on rock samples^24^,^25^. Thus we interpreted the ultrafine particles in the PSZ of both the ATTL and the Chelungpu fault as amorphous material. In contrast, the amorphous component in the surrounding intact rock and breccia around the ATTL, in which no ultrafine particles were observed (Fig. 2d,e), may be attributed to noncrystalline weathering products or damaged noncrystalline portions of large grains, because pulverization was reported within the host rocks near this outcrop^26^. The amorphous component in the intact rock around the Chelungpu fault may be attributed to volcanic glass, because the fault passes through sedimentary rocks including small amount of tuffaceous components. Although we could not definitively classify the amorphous component in the surrounding rocks, it is nevertheless significant that amorphous material and ultrafine particles were far more abundant in the PSZ of both the ATTL and the Chelungpu fault.

### Chemical kinetic evaluation of dissolution process

The central hypothesis of this study is that the ultrafine particles, produced by earthquake slip, in the PSZ may be expected to dissolve in interstitial fluids (which commonly flow along fault zones^27^,^28^) during the interseismic period, given the high solubility of mineral particles smaller than 0.1 μm^7^ and the high dissolution rate and solubility of amorphous material^7^,^8^. In the case of the Chelungpu fault, three major zones of faulting were observed in borehole samples at depths of 1136, 1194 and 1243 m^11^,^12^, but only in the shallowest of these, corresponding to the slip zone of the 1999 Chi-Chi earthquake^13^,^14^,^15^, were ultrafine particles present^6^. However, the time scale of these processes has not previously been quantified.

The dissolution rate, *k* (mol m^−2^ s^−1^), of spherical mineral particles into fluid^29^ is
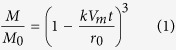
where *M* and *M*_0_ are respectively the mass and initial mass of the component, *V*_*m*_ is the molar volume of the component (m^3^ mol^−1^), *t* is time and *r*_0_ is the initial radius of the particles (m) (see Methods). The reaction rate *k* has a temperature dependency expressed by the Arrhenius equation. The kinetic data for the dissolution of representative minerals (quartz, muscovite, kaolinite and montmorillonite)^30^,^31^,^32^ and amorphous silica^8^ were experimentally determined by taking into consideration the reverse reaction (precipitation) and equilibrium state (see Methods). From these data, we evaluated the dissolution of ultrafine particles in the fault as a function of time.

We tested the time scale of the dissolution of ultrafine particles for both the ATTL and the Chelungpu fault. For the ATTL, we adopted a temperature condition of 13.8 °C (the annual mean temperature during 1979–2014 at the Sanda observatory^33^ near the outcrop) together with climatic fluctuations of ±5 °C in mean annual temperature since 47 000 years BP as reconstructed from the pollen profile from Lake Mikata^34^. Although the value of *k* also depends on thermodynamic activities of hydrogen and hydroxyl ions (that is, pH)^29^,^30^, the pH of interstitial fluid in the ATTL fault gouge was 6.0 and we therefore assumed neutral conditions (see Methods). For the Chelungpu fault, we adopted the borehole temperature of 46.5 °C determined by direct measurement^13^. Although the pH of interstitial fluid along the slip zone was not measured, it was reported that pyrite was absent in the slip zone^35^. Because pyrite dissolution would lower the pH of the immediately surrounding sediment^36^, we used pH values of 3.0 and 6.0.

By solving equation (1) and the Arrhenius equation, we determined mass fractions of each component as a function of time. The results showed that nanometric particles (100 nm radius) of kaolinite, amorphous silica and muscovite would disappear within approximately 1000 years whereas quartz and montmorillonite particles would survive longer (Fig. 4a) and that micrometric particles (10 μm radius) of all components would hardly dissolve at all within 1000 years (Fig. 4b). Given that 419 years elapsed between the 1596 Keicho-Fushimi earthquake and our sampling, some preservation of ultrafine amorphous silica particles in the ATTL fault gouge is likely, consistent with our analytical results (Fig. 2). However, the amorphous aggregates in the ATTL fault gouge were not pure silica, but also contained other atoms typical of silicate minerals (Fig. 2i). The dissolution rate at 25 °C for various silicate glasses^37^ ranges from 1.91 × 10^−9^ to 8.13 × 10^−10^ mol m^−2^ s^−1^, which is much faster than the dissolution rate of 5.59 × 10^−13^ mol m^−2^ s^−1^ for amorphous silica^8^. Silica gel, an amorphous phase found in some fault gouges^38^, has a similar dissolution rate of 9.88 × 10^−13^ mol m^−2^ s^−1^ at 18 °C^39^, and it also transforms into microcrystalline quartz by dehydration and recrystallization^40^. In the case of the Chelungpu fault, most of the nanometric particles could survive 5 years whether the pH is 3.0 or 6.0 (Fig. 4c), but micrometric particles of all components would hardly dissolve at all over 5 years (Fig. 4d). Therefore, the presence of relatively abundant ultrafine amorphous silica or silicate particles in fault material strongly suggests that the fault slipped some time in the last 1000 years.

## Discussion

Our proposed proxy requires careful consideration of certain factors. Nanometric particles are generated by breaking of grains and subsequent granulation (that is, comminution) during slip under a wide range of conditions (e.g., slip rates of 10^−9.5^ to 1.3 m s^−1^ and confining stresses of 1 to 75 MPa^24^,^25^,^41^, corresponding to depths of approximately 0.1 to 4.8 km under hydrostatic assumption with 2.6 g cm^−3^ mineral density and 1.0 g cm^−3^ pore-fluid density). Besides, generation of nanometric particles during earthquake could trigger low friction on the fault (so-called, powder lubrication)^41^,^42^. Low friction induces a large slip and a short slip weakening distance which increases both the amount of seismic energy released and the extent of the rupture area^43^.

However, nanometric particles are produced not only by slip during earthquakes but also by precipitation from cooling supersaturated fluid under geothermal conditions^44^ and by chemical reactions such as thermal decomposition of carbonate minerals (under frictional heating to approximately 600–800 °C)^45^. Surface coatings of calcite nanoparticles on fault slickensides have been also reported^46^, and these could be preserved for long periods because sintering of calcite nanoparticles occurs at room temperature by solution transfer mechanisms via adsorbed water^47^. However, such particles could be distinguished by analyzing the fluid chemistry, temperature and mineralogy of the fault material. If faulting at deeper levels caused sintering (or recrystallization) of quartz at high temperatures^48^ and the fault surface was later exposed by erosion, the sintered ultrafine quartz particles might be found in outcrops, although such particles may coalesce^49^ and recrystallise, making them distinguishable from the dispersed amorphous ultrafine particles formed near the ground surface. In addition, whereas permeable fault gouge may react during fluid flow along the fault, the dissolution reaction could be assessed by chemical equilibrium and kinetic constraints that depend on temperature, pH and solved ions (both species and strengths^8^,^37^). For amorphous silica, for example, higher temperature enhances its dissolution (Fig. 4e), and its solubility and dissolution rate are greater in alkaline conditions than in neutral or acid settings^50^,^51^ (Fig. 4f). Therefore, detailed mineralogical and chemical analyses of the fault material should also include its nanometric structure and *in-situ* environmental conditions.

The use of our proposed new proxy for assessing the recent activity of a fault may help not only to estimate the elapsed time since the most recent earthquake on a major fault, but also to characterize the activity of minor faults that may threaten important structures such as nuclear power plants, chemical plants and petroleum facilities.

## Methods

### XRD analysis

XRD spectra of the PSZ samples from the ATTL and the Chelungpu fault and their surrounding rocks were obtained with a Spectris PANalytical X’Pert PRO MPD spectrometer with monochromatized Cu Kα radiation operated at 45 kV and 40 mA, using a step width of 0.004° (∆2θ), 0.25° divergence and anti-scattering slits, and a high-speed semiconductor array detector. In addition, the samples were blended with α-alumina (25 wt.%) as an internal standard and mounted on a plate by the side-load method to minimize preferred alignment of the phyllosilicates.

### SEM and TEM observations and X-ray spectrometry

ATTL fault gouge and the surrounding hanging wall and footwall rocks were examined by a SEM (JSM-7600F, JEOL, Japan), operated at an acceleration voltage of 15 kV, and a TEM (JSM-2100, JEOL, Japan), operated at 200 kV. Elemental analyses in the SEM used energy dispersive X-ray spectrometry (Noran System 7 X-ray Microanalysis System, Thermo Fisher Scientific, USA).

### PH analysis of fault-zone fluid

Three samples of wet fault gouge along the ATTL were obtained from the outcrop (Fig. 1c). Subsamples weighing 10 g were first dried under 50 °C in an oven and then soaked in 25 mL or 50 mL of pure water. Each sample was stirred and allowed to settle for 0.5, 2 or 24 hours, and then the pH of the suspension was measured by an electrometric method^52^. The measured pH data are listed in Supplementary Table 1.

### Chemical kinetics of dissolution reactions

On the basis of the Lasaga equation expansion^29^, the amount *n* (mol) of a dissolved spherical particle is expressed as
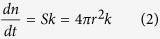
where *k* is the dissolution rate (mol m^−2^ s^−1^), *S* is the surface area (m^2^) of the particle and *r* is its radius (m). The volume decrease with time is given by
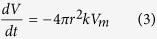
where *V* is the volume of the particle and *V*_*m*_ is the molar volume of the component (m^3^ mol^−1^, Supplementary Table 2). In terms of the radius of the sphere, the volume is also expressed as
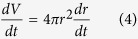
From equations (3) and (4), the decrease in size of the particle is
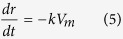
and thus,
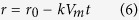
where *r*_0_ is the initial radius of the particle. Thus, the volume *V* can be expressed as
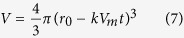
Considering the density *d* of the particle (g m^−3^) to be constant, the mass *M* of the particle is thus
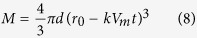
The mass fraction *M/M_0_* of the particle with time is finally obtained as
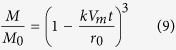
where *M*_0_ is the initial mass of the particle, and this equation is also shown as equation (1) in the Results.

The Arrhenius equation is
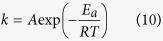
where *A* is the pre-exponential factor (mol m^−2^ s^−1^), *E*_*a*_ is the activation energy (kJ mol^−1^) and *R* is the gas constant (8.314 J K^−1^ mol^−1^). The kinetic data for dissolution of minerals (quartz, muscovite, kaolinite and montmorillonite)^30^,^31^,^32^, representative of the fault gouge, and amorphous silica^8^, experimentally determined by taking into consideration the reverse reaction (precipitation) and equilibrium state, are summarised in Supplementary Table 2.

## Additional Information

**How to cite this article**: Hirono, T. *et al.* Preservation of amorphous ultrafine material: A proposed proxy for slip during recent earthquakes on active faults. *Sci. Rep.* **6**, 36536; doi: 10.1038/srep36536 (2016).

**Publisher’s note**: Springer Nature remains neutral with regard to jurisdictional claims in published maps and institutional affiliations.

## Supplementary Material

Supplementary Information

## References

[b1] Cyranoski D. Quake shuts world’s largest nuclear plant. Nature 448, 392–393 (2007). 17653149 10.1038/448392a

[b2] Kanaori Y. Seismic risk assessment of an active fault system: the example of the Tsurugawan–Isewan tectonic line. Eng. Geol. 56, 109–123 (2000).

[b3] Cyranoski D. Quake fears rise at Japan’s reactors. Nature 494, 14–15 (2013) 23389520 10.1038/494014a

[b4] Chapman N. *et al.* Active faults and nuclear power plants. EOS 95, 33–40 (2014).

[b5] Schwartz D. P. & Coppersmith K. J. Fault behavior and characteristic earthquakes: Examples from the Wasatch and San Andreas Fault Zones. J. Geophys. Res. 89, 5681–5698 (1984).

[b6] Hirono T., Kameda J., Kanda H., Tanikawa W. & Ishikawa T. Mineral assemblage anomalies in the slip zone of the 1999 Taiwan Chi-Chi earthquake: Ultrafine particles preserved only in the latest slip zone. Geophys. Res. Lett. 41, 3052–3059 (2014).

[b7] Dove P. M. & Rimstidtm J. D. Silica-water interactions. In Silica: Physical Behavior, Geochemistry, and Materials Applications (eds Heaney P. J., Prewitt C. T. & Gibbs G. V. ) 235–290 (Rev., Mineral. 29, Mineral. Soc. Am., 1994).

[b8] Icenhower J. P. & Dove P. M. The dissolution kinetics of amorphous silica into sodium chloride solutions: Effects of temperature and ionic strength. Geochim. Cosmochim. Acta 64, 4193–4203 (2000).

[b9] Ishikawa T. *et al.* Geochemical and mineralogical characteristics of fault gouge in the Median Tectonic Line, Japan: evidence for earthquake slip. Earth, Planets, Space 66, 36 (2014).

[b10] Maruyama T. & Lin A. Active strike-slip faulting history inferred from offsets of topographic features and basement rocks: a case study of the Arima–Takatsuki Tectonic Line, southwest Japan. Tectonophysics 344, 81–101 (2002).

[b11] Hirono T. *et al.* High magnetic susceptibility of fault gouge within Taiwan Chelungpu fault: Nondestructive continuous measurements of physical and chemical properties in fault rocks recovered from Hole B, TCDP. Geophys. Res. Lett. 33, L15303 (2006).

[b12] Hirono T. *et al.* Nondestructive continuous physical property measurements of core samples recovered from hole B, Taiwan Chelungpu-Fault Drilling Project. J. Geophys. Res. 112, B07404 (2007).

[b13] Kano Y. *et al.* Heat signature on the Chelungpu fault associated with the1999 Chi-Chi Taiwan earthquake. Geophys. Res. Lett. 33, L14306 (2006).

[b14] Wu H. *et al.* Stress orientations of Taiwan Chelungpu-Fault Drilling Project (TCDP) hole-A as observed from geophysical logs. Geophys. Res. Lett. 34, L01303 (2007).

[b15] Lin W. *et al.* Current stress state and principal stress rotations in the vicinity of the Chelungpu fault induced by the 1999 Chi-Chi, Taiwan, earthquake. Geophys. Res. Lett. 34, L16307 (2007).

[b16] Chou Y.-M. *et al.* An earthquake slip zone is a magnetic recorder, Geology 40, 551–554 (2012).

[b17] Doan M. L., Brodsky E. E., Kano Y. & Ma K.-F. *In situ* measurement of the hydraulic diffusivity of the active Chelungpu Fault, Taiwan. Geophys. Res. Lett. 33, L16317 (2006).

[b18] Ma K.-F., Lin Y.-Y., Lee S.-J., Mori J. & Brodsky E. E. Isotropic events observed with a borehole array in the Chelungpu fault zone, Taiwan. Science 337, 459–463 (2012). 22837526 10.1126/science.1222119

[b19] Hirono T. *et al.* Chemical and isotopic characteristics of interstitial fluids within the Taiwan Chelungpu fault. Geochem. J. 41, 97–102 (2007).

[b20] Eberl D. D. User’s Guide to RockJock—A Program for Determining Quantitative, Mineralogy From Powder X-ray Diffraction Data. USGS Open-File Report , 03–78 (2003).

[b21] Ungar T. Microstructural parameters from X-ray diffraction peak broadening. Scr. Mater . 51, 777–781 (2004).

[b22] Marinoni N. & Broekmans M. A. T. M. Microstructure of selected aggregate quartz by XRD and a critical review of the crystallinity index. Cem. Concr. Res. 54, 215–225 (2013).

[b23] Sánchez E. C., Torres M. E., Diaz C. & Saito F. Effects of grinding of the feldspar in the sintering using a planetary ball mill. J. Mater. Process Technol. 152, 284–290 (2004).

[b24] Yund R. A., Blanpied M. L., Tullis T. E. & Weeks J. D. Amorphous material in high strain experimental fault gouges. J. Geophys. Res. 95, 15589–15615 (1990).

[b25] Hirono T. *et al.* Importance of mechanochemical effects on fault slip behavior during earthquakes. Geophys. Res. Lett. 40, 2988–2992 (2013).

[b26] Mitchell T. M., Ben-Zion Y. & Shimamoto T. Pulverized fault rocks and damage asymmetry along the Arima-Takatsuki Tectonic Line, Japan. Earth Planet. Sci. Lett. 308, 284–297 (2011).

[b27] McCaig A. M. Fluid flow through fault zones. Nature 340, 600 (1989).

[b28] Faulkner D. R. *et al.* A review of recent developments concerning the structure mechanics and fluid flow properties of fault zones. J. Struct. Geol. 32, 1557–1575 (2010).

[b29] Lasaga A. C. Chemical kinetics of water-rock interactions. J. Geophys. Res. 89, 4009–4025 (1984).

[b30] Palandri J. L. & Kharaka Y. K. A compilation of rate parameters of water-mineral intoeraction kinetics for application to geochemical modeling (U.S. Geological Survey Open File Report 2004-1068, 2004).

[b31] Nagy K. L. Dissolution and precipitation kinetics of sheet silicates. in Chemical Weathering Rates of Silicate Minerals (eds White A. F. & Brantley S. L. ) 173–233 (Rev., Mineral. 31, Mineral. Soc. Am., 1995).

[b32] Huertas F. J., Chou L. & Wollast R. Mechanism of kaolinite dissolution at room temperature and pressure, Part II: Kinetic study. Geochim. Cosmochim. Acta 63, 3261–3275 (1999).

[b33] Japan Meteorological Agency. Weather, Climate & Earthquake Information . http://www.data.jma.go.jp/obd/stats/etrn/index.php (2015).

[b34] Nakagawa T., Tarasov P. E., Nishida K., Gotanda K. & Yasuda Y. Quantitative pollen-based climate reconstruction in central Japan: application to surface and Late Quaternary spectra. Quat. Sci. Rev. 21, 2099–2113 (2002).

[b35] Hirono T. *et al.* Characterization of slip zone associated with the 1999 Taiwan Chi-Chi earthquake: X-ray CT image analyses and microstructural observations of the Taiwan Chelungpu fault. Tectonophysics 449, 63–84 (2008).

[b36] Chou Y.-M. *et al.* Pyrite alteration and neoformed magnetic minerals in the fault zone of the Chi-Chi earthquake (Mw 7.6, 1999): Evidence for frictional heating and co-seismic fluids. Geochem. Geophys. Geosyst. 13, Q08002 (2012).

[b37] Icenhower J. P. *et al.* Experimentally determined dissolution kinetics of Na-rich borosilicate glass at far from equilibrium conditions: Implications for Transition State Theory. Geochim. Cosmochim. Acta 72, 2767–2788 (2008).

[b38] Goldsby D. L. & Tullis T. E. Low frictional strength of quartz rocks at subseismic slip rates. Geophys. Res., Lett. 29, 1844 (2002).

[b39] Rimstidt J. D. & Barnes H. L. The kinetics of silica-water reactions. Geochim. Cosmochim. Acta 44, 1683–1699 (1980).

[b40] Faber C., Rowe C. D., Miller J. A., Fagereng A. & Neethling J. H. Silica gel in a fault slip surface: Field evidence for palaeo-earthquakes? J. Struct. Geol. 69, 108–121 (2014).

[b41] Han R., Hirose T., Shimamoto T., Lee Y. & Ando J. Granular nanoparticles lubricate faults during seismic slip. Geology 39, 599–602 (2011).

[b42] Reches Z. & Lockner D. A. Fault weakening and earthquake instability by powder lubrication. Nature 467, 452–455 (2010). 20865001 10.1038/nature09348

[b43] Kanamori H. & Brodsky E. E. The physics of earthquakes. Rep. Prog. Phys. 67, 1429–1496 (2004).

[b44] Tobler, D., J. & Benning L. G. *In situ* and time resolved nucleation and growth of silica nanoparticles forming under simulated geothermal conditions. Geochim. Cosmochim. Acta 114, 156–168 (2013).

[b45] Han R., Shimamoto T., Hirose T., Ree J.-H. & Ando J. Ultralow friction of carbonate faults caused by thermal decomposition. Science 316, 878–881 (2007). 17495168 10.1126/science.1139763

[b46] Siman-Tov S., Aharonov E., Sagy A. & Emmanuel S. Nanograins form carbonate fault mirrors. Geology 41, 703–706 (2013).

[b47] Verberne B. A., Plümper O., Matthijs de Winter D. A. & Spiers C. J. Superplastic nanofibrous slip zones control seismogenic fault friction. Science 346, 1342–1344 (2014). 25504714 10.1126/science.1259003

[b48] Suzdal’tsev E. I. The sintering process of quartz ceramics. Refractories and Industrial Ceramics 44, 236–241 (2003).

[b49] Koizumi S. *et al.* Synthesis of highly dense and fine-grained aggregates of mantle composites by vacuum sintering of nano-sized mineral powders. Phys. Chem. Minerals 37, 505–518 (2010).

[b50] Alexander G. B., Heston W. M. & Iler R. K. The solubility of amorphous silica in water. J. Phys. Chem. 58, 453–455 (1954).

[b51] Niibori Y., Kunita M., Tochiyama O. & Chida T. Dissolution rates of amorphous silica in highly alkaline solution. J*. Nucl. Sci. Tech.* 37, 349–357 (2000).

[b52] Pansu M. & Gautheyrou J. Handbook of Soil Analysis (Springer-Verlag, 2006).

[b53] Earthquake Research Committee, Headquarters for Earthquake Research Promotion, Ministry of Education, Culture, Sports, Science and Technology, Japan. National Seismic Hazard Maps for Japan . http://www.jishin.go.jp/main/chousa/06mar_yosoku-e/NationalSeismicHazardMaps.pdf (Data of access: 20 January 2016) (2005).

