# Supplementary material for: Preservation of amorphous ultrafine material: A proposed proxy for slip during recent earthquakes on active faults
Source: Sci Rep. 2016 Nov 9;6:36536. doi: 10.1038/srep36536 (PMC5101524; doi:10.1038/srep36536)
Supplement: Supplementary Information [file srep36536-s1.pdf]

SUPPLEMENTARY INFORMATION

Preservation of amorphous ultrafine material: A proposed proxy for slip during recent earthquakes on active faults

Tetsuro Hirono[1*], Satoru Asayama[1], Shunya Kaneki[1] and Akihiro Ito[2]

[1] Department of Earth and Space Science, Graduate School of Science, Osaka University, Toyonaka, Osaka 560-0043, Japan.

[2] Analytical Instrument Facility, Graduate School of Science, Osaka University, Toyonaka, Osaka 560-0043, Japan.

* Corresponding author. Contact: hirono@ess.sci.osaka-u.ac.jp

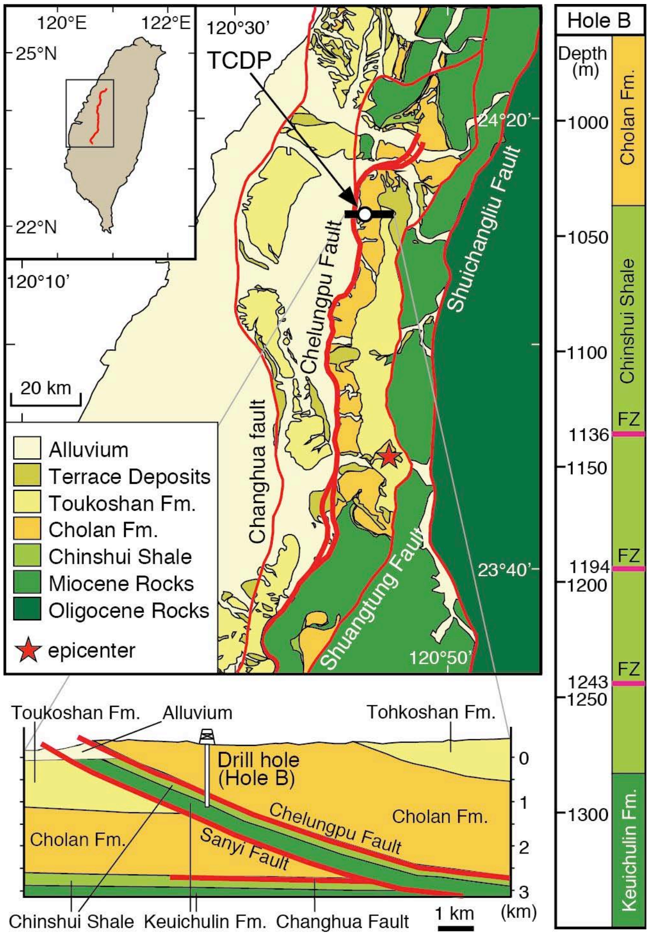

Supplementary Figure 1. The Taiwan Chelungpu-fault Drilling Project (TCDP). Geological map of central Taiwan showing the drilling site, an E-W cross section through the site and the three dominant fault zones discovered at the depths of 1136 m1194 m and 1243 m at Hole B. This figure was reused from our previous published data[54]. The shallowest fault zone at 1136 m in depth was most likely the one that slipped during the 1999 Chi-Chi earthquake, explained in the main text. FZ, fault zone; Fm., Formation.

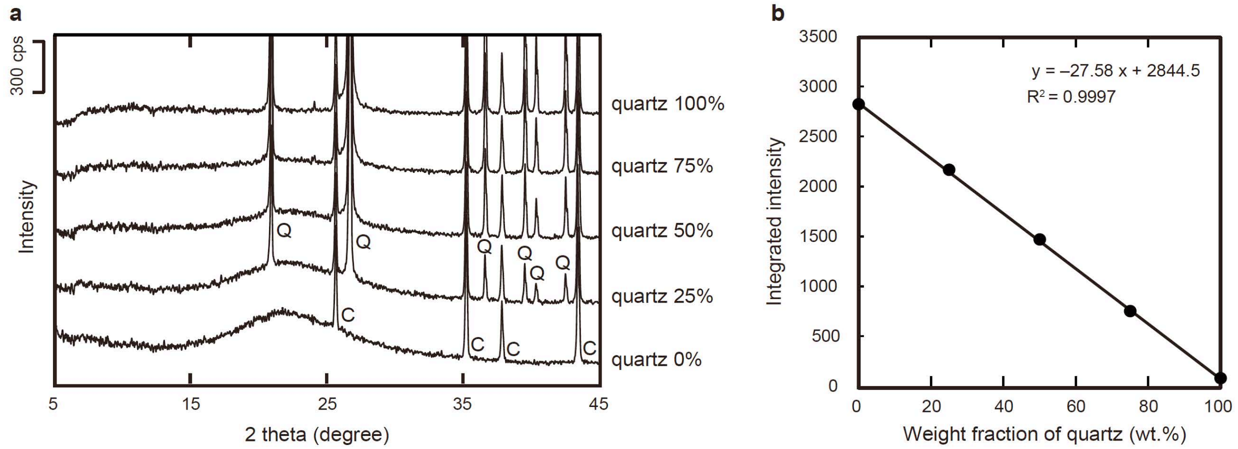

Supplementary Figure 2. Quantification of amorphous component. a, XRD patterns for different mixtures of quartz and amorphous silica. Q, quartz; C, corundum (α-alumina). **b,** Weight fraction of quartz versus integrated intensity of the broad bump.

Supplementary Table 1. Measurements of pH in the ATTL fault gouge

Fault sample number	Ratio of sample and water	Suspension duration (hour)	pH
PSZ1	1:25	0.5	6.0
PSZ1	1:25	2.0	6.0
PSZ1	1:25	24.0	5.9
PSZ1	1:5	0.5	5.9
PSZ1	1:5	2.0	6.0
PSZ1	1:5	24.0	6.0
PSZ2	1:25	0.5	6.1
PSZ2	1:25	2.0	6.0
PSZ2	1:25	24.0	5.9
PSZ2	1:5	0.5	6.0
PSZ2	1:5	2.0	6.0
PSZ2	1:5	24.0	5.9
PSZ3	1:25	0.5	6.0
PSZ3	1:25	2.0	6.0
PSZ3	1:25	24.0	5.9
PSZ3	1:5	0.5	6.0
PSZ3	1:5	2.0	6.0
PSZ3	1:5	24.0	5.9
**Average**	–	**–**	**6.0**

Supplementary Table 2. Kinetic parameters for dissolution of mineral components

Materials	k at 25 °C (mol m^{-2} s^{-1})	A value (mol m^{-2} s^{-1})	E_a value (kJ)	Calculated k at 13.8 °C (mol m^{-2} s^{-1})	Calculated k at 18.8 °C (mol m^{-2} s^{-1})	Calculated k at 8.8 °C (mol m^{-2} s^{-1})	Calculated k at 46.5 °C (mol m^{-2} s^{-1})	V_m (m^3 mol^{-1})
Quartz	3.98×10^{-14}	3.3×10^{2}	90.9	9.33×10^{-15}	1.82×10^{-14}	4.79×10^{-15}	4.57×10^{-13}	2.22×10^{-5}
Amorphous SiO_2	5.89×10^{-13}	6.7	74.5	1.82×10^{-13}	3.09×10^{-13}	1.05×10^{-13}	4.47×10^{-12}	2.73×10^{-5}
Muscovite	2.82×10^{-14}	2.0×10^{-10}	22.0	2.00×10^{-14}	2.34×10^{-14}	1.70×10^{-14}	5.13×10^{-14}	1.37×10^{-4}
Muscovite (pH=3.0)	1.41×10^{-12}	1.0×10^{-8}	22.0				1.99×10^{-13}	1.37×10^{-5}
Kaolinite	6.61×10^{-14}	5.1×10^{-10}	22.2	4.68×10^{-14}	5.50×10^{-14}	3.98×10^{-14}	1.20×10^{-13}	1.99×10^{-4}
Montmorillonite	3.89×10^{-14}	1.0×10^{-5}	47.0	1.82×10^{-14}	2.57×10^{-14}	1.29×10^{-14}	1.41×10^{-13}	9.09×10^{-4}
Montmorillonite (pH=3.0	1.95×10^{-13}	2.7×10^{-9}	23.6				8.09×10^{-14}	9.09×10^{-5}

Supplementary References:

54. Maekawa, Y. *et al.* Estimation of slip parameters associated with frictional heating during the 1999 Taiwan Chi-Chi earthquake by vitrinite reflectance geothermometry. *Earth Planets Space* **66**:28 (2014).
